# The TROLLEY Study: assessing travel, health, and equity impacts of a new light rail transit investment during the COVID-19 pandemic

**DOI:** 10.1186/s12889-022-13834-1

**Published:** 2022-08-02

**Authors:** Katie Crist, Tarik Benmarhnia, Lawrence D. Frank, Dana Song, Elizabeth Zunshine, James F. Sallis

**Affiliations:** 1 Department of Urban Studies & Planning, UC San Diego, 9500 Gilman Drive, La Jolla, San Diego, CA 92093 USA; 2 Scripps Institution of Oceanography, UC San Diego, 9500 Gilman Drive, La Jolla, San Diego, CA 92093 USA; 3 Moores Cancer Center, UC San Diego, 9500 Gilman Drive, La Jolla, San Diego, CA 92093 USA; 4 Herbert Wertheim School of Public Health and Human Longevity Science, UC San Diego, 9500 Gilman Drive, La Jolla, San Diego, CA 92093 USA; 5 Mary MacKillop Institute for Health Research, Australian Catholic University, Melbourne, Australia

**Keywords:** Physical activity, Transportation, Light rail transit, Active transportation, Built environment, Workplace, Accelerometer, GPS, Active travel

## Abstract

**Background:**

The COVID-19 pandemic disrupted life in extraordinary ways impacting health and daily mobility. Public transit provides a strategy to improve individual and population health through increased active travel and reduced vehicle dependency, while ensuring equitable access to jobs, healthcare, education, and mitigating climate change. However, health safety concerns during the COVID-19 pandemic eroded ridership, which could have longstanding negative consequences. Research is needed to understand how mobility and health change as the pandemic recedes and how transit investments impact health and equity outcomes.

**Methods:**

The TROLLEY (TRansit Opportunities for HeaLth, Livability, Exercise and EquitY) study will prospectively investigate a diverse cohort of university employees after the opening of a new light rail transit (LRT) line and the easing of campus COVID-19 restrictions. Participants are current staff who live either < 1 mile, 1–2 miles, or  > 2 miles from LRT, with equal distribution across economic and racial/ethnic strata. The primary aim is to assess change in physical activity, travel mode, and vehicle miles travelled using accelerometer and GPS devices. Equity outcomes include household transportation and health-related expenditures. Change in health outcomes, including depressive symptoms, stress, quality of life, body mass index and behavior change constructs related to transit use will be assessed via self-report. Pre-pandemic variables will be retrospectively collected. Participants will be measured at 3 times over 2 years of follow up. Longitudinal changes in outcomes will be assessed using multilevel mixed effects models. Analyses will evaluate whether proximity to LRT, sociodemographic, and environmental factors modify change in outcomes over time.

**Discussion:**

The TROLLEY study will utilize rigorous methods to advance our understanding of health, well-being, and equity-oriented outcomes of new LRT infrastructure through the COVID-19 recovery period, in a sample of demographically diverse adult workers whose employment location is accessed by new transit. Results will inform land use, transportation and health investments, and workplace interventions. Findings have the potential to elevate LRT as a public health priority and provide insight on how to ensure public transit meets the needs of vulnerable users and is more resilient in the face of future health pandemics.

**Trial registration:**

The TROLLEY study was registered at ClinicalTrials.gov (NCT04940481) June 17, 2021, and OSF Registries (10.17605/OSF.IO/PGEHU) June 24, 2021, prior to participant enrollment.

**Supplementary Information:**

The online version contains supplementary material available at 10.1186/s12889-022-13834-1.

## Background

Reducing health inequalities and harms from physical inactivity and air pollution are UN priorities [1, 2]. Public transit provides an opportunity to address these major challenges by increasing physical activity (PA) through active transportation (AT), mitigating greenhouse gas (GHG) emissions by decreasing vehicle miles traveled (VMT), and providing equitable access to jobs, education, and healthcare. Safe and accessible transit is critical for low-income groups. Essential workers comprise 26% of the working age population and 40% of transit riders in the U.S. [3], and nearly 50% are people of color [4, 5]. Given that more than 80% of the U.S. population lives in urban areas, LRT has potential to impact a majority of the population and to deliver numerous co-benefits [6, 7].

Sufficient PA is associated with reduced risk of numerous chronic conditions, including cardiovascular disease (CVD), type 2 diabetes, cancer, stroke, hypertension, and premature mortality [8–11]. Regular PA is additionally linked with improved well-being, fewer depressive symptoms [12], less stress [13–15], and improved quality of life (QoL) [16–19]. Despite known health benefits, fewer than half of U.S. adults meet the recommendation of 150 min/wk of PA [10, 11, 20, 21]. Additionally, there are PA disparities by income and race/ethnicity [22–28]. While the prevalence of sufficient leisure time PA is stable or increasing in white and college-educated populations, minority, less-educated and low-income groups are experiencing declines and have lower PA levels overall [29, 30]. Transit use often involves walking or biking to stations or destinations and is associated with more minutes of PA and meeting PA guidelines [31–38], providing an approach to combat physical inactivity [39–41]. Furthermore, promoting active transportation helps reduce greenhouse gas and traffic-related air pollution emissions which offers substantial co-benefits [42].

Americans in the lowest income quintile spend 1/3^rd^ of their income on transportation, a proportion that decreases as income rises [43]. These costs can impact spending on health-related expenses. Low-income households are more likely to have difficulty purchasing healthy foods [44, 45] and are less likely than higher-income households with similar health needs to access medical care [46]. Low-income households are expected to experience the greatest financial benefit from reducing the costs of vehicle ownership by replacing car trips with transit [47]. Evidence of the benefit of LRT access on employment status in low-income workers has been mixed [48, 49]. The Moving to Opportunity evaluation found that, while improved transit access was not associated with new employment, the odds of *maintaining* employment 4 to 7 years later was higher for those with better transit access [50, 51].

Transit ridership dropped sharply during COVID-19 due to health concerns. Studies have shown the greatest shift in travel mode was from public transit to private vehicles [52, 53]. The declines in transit use were smallest in communities with lower socioeconomic status and a higher proportion of essential workers [54, 55]. These findings highlight the vital role transit serves not only for essential workers, but for the greater population dependent on the essential services they provide [56].

The TROLLEY (TRansit Opportunities for HeaLth, Livability, Exercise and EquitY) study will prospectively quantify changes in multiple travel, health, and equity outcomes in a sample of diverse, adult workers following the opening of a new LRT line nearly two years into the COVID-19 pandemic. This research is uniquely generalizable within the post pandemic period. It further provides an advancement beyond prior LRT evaluations by recruiting employees who both live and work near rail stations [57]. The study provides a timely assessment of the impact of COVID-19 on transit use and perceptions and whether behaviors and health outcomes vary by socioeconomic and built environment factors.

## Methods/Design

### TROLLEY Study Design and Setting

TROLLEY is a prospective cohort study of 465 adults working at UC San Diego (UCSD) at 3 time points over 2 years of follow up. The new 11-mile UC San Diego Blue Line light rail extension opened in November 2021. This new line connected transit-served low-income and diverse communities in south and east San Diego County, where a large proportion of UCSD’s roughly 18,000 staff reside, to the major employment, education, and healthcare center of UCSD. The trolley line was completed prior to participant enrollment. However, data on travel, health and spending behaviors before COVID-19 and the LRT line opening will be collected retrospectively to allow for a pre-post comparison. UCSD is San Diego County’s second largest employer with a majority of staff from racial or ethnic minority groups and 90% filling non-managerial roles [58]. The new LRT line has three stops accessing the UCSD La Jolla campus and the VA and UCSD medical centers, providing a functional transportation option for university and healthcare workers. Unlike previous LRT intervention studies that recruited people around new stations that may or may not connect them with employment destinations [59–66], we will recruit staff who work near LRT and live either within 1 mile, 1–2 miles, or  > 2 miles from any San Diego LRT station. Previous research has long shown that environmental features at both home and work locations are significantly related with travel mode choice [67]. Therefore, the sampling design we proposed has greater potential to capture the impact on commute travel patterns.

**Fig. 1:**
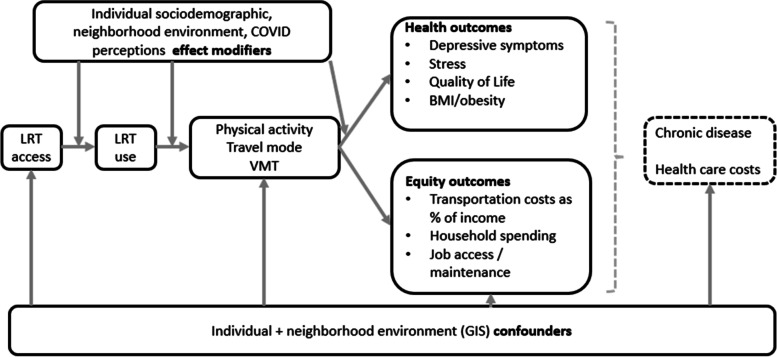
TROLLEY Study Conceptual Model

The TROLLEY study framework, summarized in Fig. 1, is based on multilevel ecological models that highlight the many levels of influence on active living behaviors [68].

Our study will assess selected variables at multiple ecological levels to understand how LRT access, demographics, perceptions/beliefs, travel demand programs, and built environment factors explain travel behaviors and, in turn, health and equity outcomes. Anticipated longer-term chronic disease impacts, shown elsewhere to be related with travel-related PA [69, 70], and downstream healthcare costs will not be assessed in this study due to the 2-year time frame.

The study was approved by UC San Diego’s Institutional Review Board prior to participant enrollment (Protocol #804110). All protocol modifications will be approved by the investigative team and submitted to the IRB. Participants consent to data being used in future analyses.

### Study Aims

The primary aim is to evaluate change in device-measured total and moderate-to-vigorous PA (MVPA), travel mode, and VMT. We expect minutes of MVPA and the proportion of AT (bike, walk, transit) trips, as a share of total trips, will increase. We also expect vehicle trips and VMT will decrease over time in those who live closest to LRT stations, compared to those who have less access to LRT.

### Equity aims

We will assess change in household expenditures, including transportation costs as a proportion of household income. We hypothesize the proportion of household income spent on transportation costs will decrease for those who utilize the new LRT, allowing for increased spending on health and QoL-related expenditures (i.e., healthy foods, medications, healthcare, etc.). We expect those with lower household income will have the largest decrease in transportation costs as a proportion of household income. We will additionally assess whether transit use is associated with an increase in the proportion of new UCSD hires from low-income areas and people of color, as well as potential impacts on employment continuity. 

### Secondary health aims

We will measure change in secondary health outcomes, including depressive symptoms, stress, QoL, and body mass index (BMI). We hypothesize those who use LRT will have fewer depressive symptoms, lower stress, greater QoL, and less weight gain over time than non-users.

### Transit and COVID-19 perception aims

We will evaluate transit-related intentions, benefits, barriers, social support, satisfaction, and self-efficacy in both transit riders and non-riders. We will track COVID-19 infection and vaccination status, and we expect transit and COVID-19 perceptions and infection/vaccination status to be independently related to LRT use.

### Subgroup analyses (effect modifiers)

We will examine whether change in outcomes over time is modified by distance to LRT, sociodemographic, and neighborhood environment (i.e., walkability) variables. We hypothesize those living closer to LRT stations, living in more walkable neighborhoods, or accessing more walkable stations, and low-income staff will improve their health and economic outcomes to a greater extent.

### Study sample and eligibility

Prior evaluations of new LRT have recruited people into treatment (exposed) and control groups based on distance from their homes to new rail stations [59–66]. There is debate on the degree to which LRT investments induce new transit ridership versus shifting existing users from bus to rail [57, 71–73]. The current study attempts to overcome this limitation by focusing recruitment on university staff who both live and work near the rail line. We are not recruiting students, given the longitudinal design, nor physicians/faculty, whose level of education and income would be higher than average and not generalizable to the broader population. Compared to faculty or students, UCSD staff live twice the average distance from campus, and roughly 50% live in areas accessed by transit [74]. To be eligible, participants must be 18 years of age or older, be a full or part-time employee at the UCSD La Jolla campus, have commuted to the La Jolla campus ≥ 2 days/wk prior to COVID-19 closures, have lived in the same location for at least 1 year with no plans to move, can walk without assistance, can read and write in English or Spanish, and spend most of their time in San Diego County. We aim to have equal distribution across distance to LRT groups (< 1 mile, 1–2 miles, > 2 miles), with 50% of the sample comprised by females, people of color, and low-income staff.

### Recruitment

#### Home location catchment areas

**Fig. 2:**
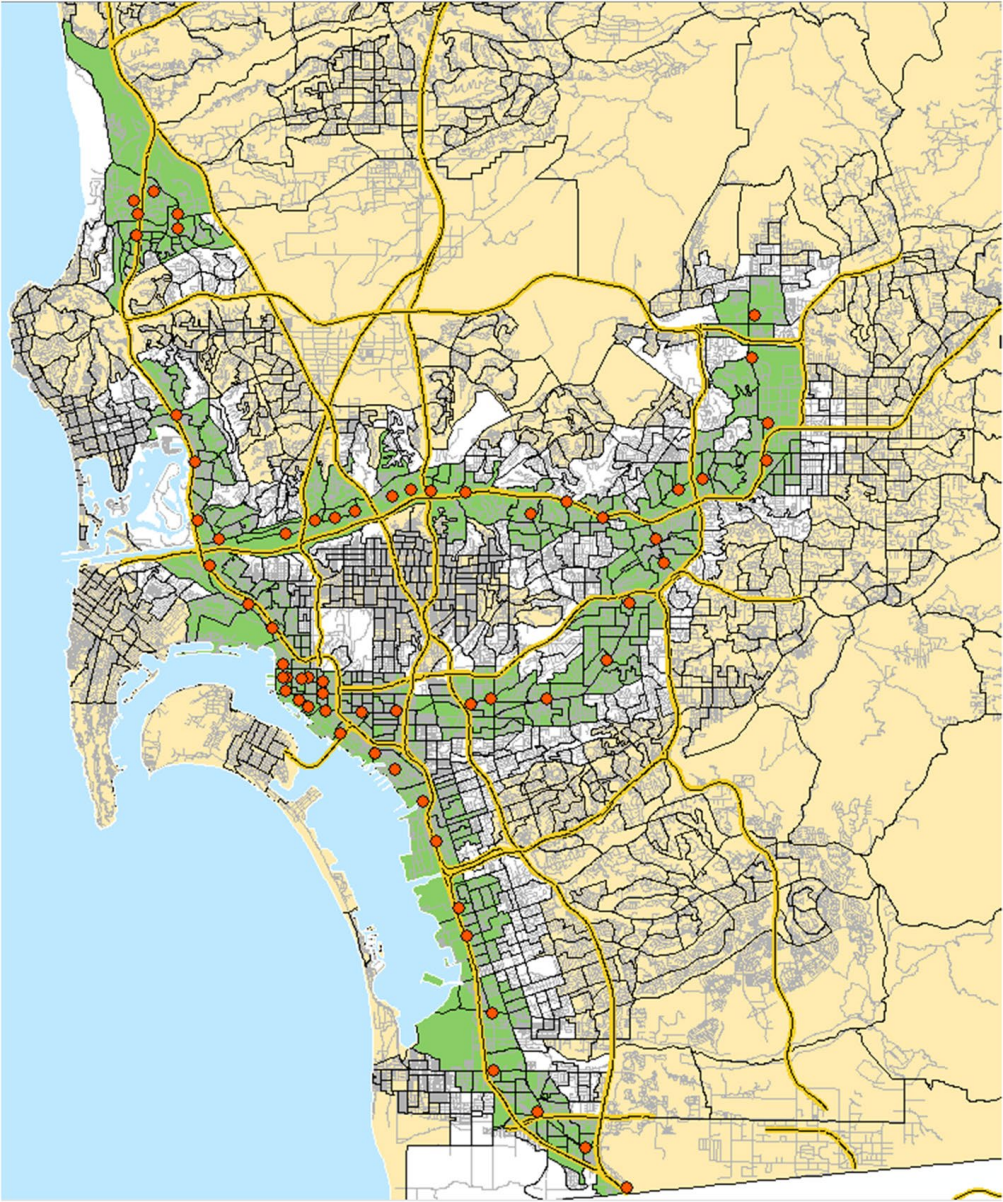
LRT stations with recruitment catchment areas MTS LRT stations (red), census block groups within one mile with at least 25% area overlap with station areas (green), block groups between one and two miles (white), block groups greater than 2 miles (no-LRT-group) with at least 25% area overlap with two-mile catchment areas (beige). Figure created by authors

The recruitment groups (< 1-mile, between 1 and 2 miles, and > 2 miles) were selected to ensure variability in distance to LRT from participants’ home location. We consider the 2 + mile group to be unexposed, as research shows few people in the US travel further than 2 miles by bike to reach transit [75–77]. We identified walk catchment areas within the described network distances from any Metropolitan Transit System (MTS) trolley station (i.e., the Blue line or any connecting trolley line). Since pedestrians travel along transportation networks, we used network-based catchment areas which have a much more accurate representation of urban form features accessible within a given walk distance. A “sausage” or balloon network buffer was developed to define all areas within the catchment areas with a 25-m (82 feet) trim or setback from the roadway. This expanded polygon intersects with parcels and land uses that front on the selected road segments so that homes and apartment buildings could be spatially matched to the buffers. Each catchment area was intersected with census block groups to determine the percentage of block groups contained. Block groups with less than 25% of their area in the buffer were not included. Figure 2 shows the LRT stations and recruitment catchment areas.

The primary recruitment strategy is to identify interested and eligible staff through a pre-screening survey invitation sent by UCSD’s Department of Resource, Management and Planning, in both English and Spanish. The email was sent to > 17,000 active UCSD career staff, identified by their job title code. This method allows us to target staff specifically, exclusive of students and faculty. The email contains an embedded link to the study screener from our secure study database. Those who choose to complete the pre-screening survey provide their contact information, home address or nearest intersection, and answer the eligibility screening questions. The home addresses of staff who meet the initial screening criteria are then geocoded and spatially joined to the recruitment catchment buffers to determine which catchment area they live in. Those who are not eligible receive an email and a phone call explaining the reasons for ineligibility. Those who meet eligibility criteria are contacted by research staff who confirm interest and eligibility, answer questions, and obtain written informed consent.

In addition to university email, we are recruiting through UCSD staff distribution listservs and flyers, staff associations and unions, and on-campus advertising. These recruitment efforts target key UCSD units like Housing, Dining and Hospitality services, the Black, Chicanx/Latinx, and Pan-Asian Staff Associations, and hospital nursing and support staff groups to reach the intended population. Recruitment began in January 2022 and is ongoing, with 230 participants currently enrolled.

### Survey testing sample

A sample of 40 UCSD staff was recruited to test the reliability of the study survey. After pre-testing near-final drafts, we evaluated the test–retest reliability and internal consistency of new survey items by administering it twice, roughly 2 weeks apart in a sample of 40 transit users and non-users in both Spanish and English. Participants met the same eligibility criteria as the full study sample.

### Sample size

The TROLLEY study will enroll 465 English or Spanish speaking staff from UCSD’s La Jolla campus. We estimated a sample size of 340 was needed, considering a baseline of 45.1 (SD: 4.6) minutes/week of moderate PA using NHANES (2005–06) adult accelerometer data. We used a meta-analysis by Hirsh et al. and a conservative expected change of 3.46 min/day (95%CI: 2.20, 4.72) in transit-related PA from Miller et al. that evaluated a similar intervention in Utah [62, 78]. We set a significance level of 0.05 and power levels of 95% and assumed a 20% attrition rate. However, to ensure sufficient power for planned moderator analyses and in case of higher attrition rates than planned, we will recruit a total sample of 465 participants, stratified by LRT exposure, income, and race/ethnicity.

### Data collection

Participants will complete a measurement visit after consenting to participation. Participants will be assessed 3 times during the 2 years they are enrolled in the study; once when first enrolled (2022), 1 year after enrollment (2023), and 2 years after enrollment (2024). All data collection will occur after the opening of the UCSD Blue Line Trolley. However, time 1 survey measures ask participants about commute, PA, and spending behaviors both at the time of assessment and, retrospectively, prior to the pandemic and the new LRT. The campus closure in March of 2020 due to COVID-19 provides a noteworthy reference point to aid recall. The UCSD Exercise and Physical Activity Resource Center (EPARC) distributes accelerometer and GPS devices and instructs participants how to wear the monitors. TROLLEY staff call participants during the wear week to ensure compliance. Links to the online surveys are emailed to participants, and participants receive a $50 gift card after completion of all assessments at each time point.

Study data are collected and managed using REDCap electronic data capture tools hosted at UC San Diego [79]. REDCap (Research Electronic Data Capture) is a secure, web-based application designed to support data capture for research studies, providing: 1) an intuitive interface for validated data entry; 2) audit trails for tracking data manipulation and export procedures; 3) automated export procedures for seamless data downloads to common statistical packages; and 4) procedures for importing data from external sources. Participants are assigned a study ID and all records are coded with the study ID rather than personal identifiers. All device data files will be kept on secure servers at the Social Sciences Computing Facility and data will only be accessible to investigators and project staff.

### Device measured outcomes

The primary outcomes are total PA and MVPA minutes per day, travel mode, and VMT. We will employ two best-available technologies, accelerometer and GPS devices, to objectively measure PA and location. These devices allow us to 1) precisely measure change in PA minutes and to specifically attribute any change in PA to LRT trips, and 2) determine travel mode to assess change in commute mode and VMT. 

### Minutes of MVPA

We will use Actigraph GT3X + accelerometers and process the data with Actilife software (ActiGraph, LLC; Pensacola, FL). Participants are asked to wear the device on a belt on their hip during waking hours for 7 days at each measurement point. Participants are asked to re-wear the devices if not worn for at least 10 h per day for 4 days to ensure data are representative of habitual PA [80–82]. We will use the established cut off of 1952 counts per minute (CPM) to determine minutes of MVPA during analysis [83]. To provide perspective on PA disparities reduction, we will assess the % of participants who meet PA guidelines of 150 min of MVPA per week.

### Travel mode share and VMT

GPS devices will be used to determine PA location, trip mode and trip distance. Participants will wear the Qstarz BT1000x GPS device (Qstarz International Co. Ltd., Taipei, Taiwan) to log X,Y location coordinates, distance, speed, elevation and time. The Qstarz GPS is smaller than a cell phone and is worn inside a pouch on the same belt as the accelerometer. Participants must charge this device every evening, and we have developed protocols to maximize adherence. The device captures data every 15 s and has an industry-reported accuracy of 3 m, with validation studies showing a median error of 2 m for bike and 3.9 m for walk trips [84]. We will use a validated imputation algorithm for missing data points [85]. After consideration of non-wear time and missing data, valid wear days of 10 h or more will be merged with the accelerometer data by time stamp at the minute level, using the HABITUS (Human Activity Behavior Identification Tool and data Unification System) [86]. HABITUS is a web-based service that processes these data to determine transportation trips, mode, routes, and distances travelled. VMT will be assessed using these data and self-reported vehicle odometer readings.

### Self-report measures and covariates

#### Equity Outcomes

Household expenditures and transportation costs will be assessed by self-report using survey measures from the Health and Retirement Study [87]. The Consumption and Activities Mail Survey captures information about healthcare, food, and transportation expenditures. The Health Care and Nutrition Survey considers whether health care access and nutrition spending were impacted by income (e.g., “In the last 12 months, did you ever eat less that you felt you should because there wasn’t enough money for food?”). All participants will be employed at baseline, but we will ask about changes in employment at all future assessments. Secondary human resource data will capture summary measures of new employees during the project period to determine the impact of the LRT line on employment opportunities (for example, a summary measure of the percentage of new employees using LRT as their primary commute method). Health care access and utilization will be assessed using questions from the Behavioral Risk Factor Surveillance System (BRFSS) survey.

Secondary health outcomes will be evaluated using validated self-report measures. We will use the 10-item Center for Epidemiological Studies-Depression Scale (CES-D Short-form), which is a commonly used population-based scale for measuring depressive symptoms in adults [88–90]. The 20-item Perceived Quality of Life scale (PQoL-20) asks participants to rate their satisfaction with various aspects of life [91], and the SF-12 Health Survey will measure health-related QoL [92, 93]. The Cohen Perceived Stress Scale measures the degree to which situations in one’s life are perceived as stressful [94]. To better understand sources of stress from discrimination and racism in our cohort, we included an additional measure of stress. The validated Everyday Discrimination Scale asks how often participants experience discrimination in their daily life based on their race, ethnicity or skin color [95–97]. The NIH PROMIS Sleep Disturbance 6a Short Form will assess perceptions of sleep quality, sleep depth, and restoration [98]. The Global Physical Activity Questionnaire allows us to capture self-reported PA in the occupational, transportation, and leisure domains [99, 100]. Participants will self-report height and weight, and BMI will be calculated (kg/m^2^) [101].

### Transit beliefs and perceptions

We developed and evaluated a survey based on theory-driven behavior change determinants, adapted to relate specifically to LRT use, to understand how attitudes and perceptions shift over time after campus reopened from COVID-19 closures. Using the Social Cognitive Theory [102] and Transtheoretical Model [103], survey measures assess the following constructs: intentions to use LRT, benefits (e.g. health, cost, climate) and barriers to use (e.g. access, schedules, safety), social support (e.g. family/coworkers use or encourage LRT use), satisfaction with transit service (e.g. hours, frequency, access, stops) and COVID-19 safety measures (e.g. ventilation systems, touchless pay stations, sanitization procedures), self-efficacy (e.g. confidence in ability to use transit safely), outcome expectancy (e.g. effectiveness of COVID-19 mitigation measures), and COVID-19 infection and vaccination status.

### Covariates and effect modifiers

Sociodemographics include age, sex, race/ethnicity, education, marital status, annual household income, number of adults and children in the household, vehicle access, driving status, caretaking/childcare responsibilities, and years living at address. Health Status will be measured with a subset of the NHANES ‘medical conditions’ survey [104]. An 18-item questionnaire that measures reasons for moving to your neighborhood will be used to adjust for residential self-selection, which is a potential bias in environmental studies [105].

### Environmental effect modifiers

We will use the US Environmental Protection Agency’s Smart Location Database (SLD) of key built and social environment variables for all census block groups and the areas around LRT stations [106, 107]. These GIS data include variables for: Density (population, employment, housing, etc.); Diversity (mixture of uses, incomes, etc.); Design (walkability/intersection density), and Destination Accessibility (number of jobs accessible by transit and auto). Proximity to the trolley line of residential and occupational addresses will be calculated using GIS from geocoded locations. Neighborhood perceptions around home locations will be measured by self-report using the validated and widely used Neighborhood Environment Walkability Scale (NEWS)—Abbreviated survey [108–110] which has been associated with AT in adults [111].

### Statistical approach

Summary statistics of individual-level demographics, census block attributes, and baseline values of all outcomes will be calculated for the full sample and compared between the LRT exposure groups. Variables that are not balanced across groups will be adjusted for in subsequent analyses. Daily minutes of MVPA are typically positively skewed and will be transformed as needed to better approximate normality. Longitudinal changes in outcomes across the 3 measurement time points will be assessed using multilevel mixed effects linear (for continuous outcomes) or multinomial or ordinal logistic (for categorical outcomes) regression models, with the measurement time point included as an independent variable and adjustment for individual or block group level covariates (i.e., demographic characteristics, walkability, etc.). Covariates will be selected using a priori-knowledge and Directed Acyclic Graphs. Each outcome will be modeled separately. As we will have multiple measurement days nested within participants, a random participant-level intercept will be included in all models (alternative approaches that consider such clustering based on generalized estimation equations (GEE) will be also tested as a sensitivity analysis). We will also test the extent of block group clustering for each outcome (without covariates) by including a random effect for the intercept at the block group level and estimating the intra-class correlation (ICC). A likelihood-ratio test will assess fit between the models with and without clustering and, if significant, a random effect for block group will be included. We will assess effect modification by LRT exposure and other variables on both additive and multiplicative scales (when focusing on binary, categorical or count outcomes) using traditional approaches (stratified analyses coupled with heterogeneity tests or by including an interaction term in our models) [112, 113].

We will perform additional sensitivity analyses to maximize covariate balance between LRT groups, considering techniques like inverse probability of treatment weighting (IPTW). IPTW removes confounding by creating a pseudo population in which every participant has an equal probability of being in the LRT- exposed group (i.e., home < 1 mile from LRT stop), assuming no unmeasured confounding. We will first model participants’ probability (i.e. the propensity score (PS) of being in the LRT-exposed group) [114] using imbalanced baseline demographic and environmental characteristics [115–117]. We will then calculate a weight using the PS values that will be included in regression models. Estimates from weighted and unweighted models will be compared and will also implement stabilized and truncated weights. We will examine the extent of missing data and conduct sensitivity analyses to compare characteristics of participants with complete versus incomplete data. Multiple imputation methods will be applied, assuming missing-at-random patterns and using default multiple imputation models (“mice” in R), with *N* imputed data sets generated (*N* corresponding the average % of missingness).

Test–retest reliability of the transit and COVID-19 survey measures, with two weeks in between assessments, will be assessed using intraclass correlation coefficients (ICC) [118, 119]*.* Values above 0.75 will be considered good to excellent reliability.

## Discussion

The TROLLEY study has a unique opportunity to quantify change in key travel, health, and equity-related outcomes associated with the introduction of new LRT infrastructure and in the context of the COVID-19 pandemic. Research is needed to explore variables that may prevent increased vehicle dependence as a long-term consequence of the pandemic [53], and inform interventions to improve individual and climate health indicators. Despite promising cross-sectional evidence of associations between LRT and health, including MVPA, longitudinal evidence is less consistent [31, 36, 38, 120–122]. The TROLLEY study has the potential to document a more powerful effect than previous LRT evaluation studies based on the unique sampling of employees whose work location is newly accessed by LRT and who live near an LRT station. Low-income communities bear a greater burden from transportation costs and reliance on vehicles that negatively impacts health. Improving transit access may provide a cost-effective and long lasting approach to improve PA and health, with broad reach to lower income employees [123]. The TROLLEY study will improve our understanding of the mobility needs of vulnerable groups and transit as a workplace health and equity strategy.

The 2-year length of the study presents a challenge to participant retention. While we have planned for 20% attrition, the university sample allows more opportunity to maintain contact with participants and mitigate loss to follow up. The lack of accelerometer and GPS device data prior to the opening of the new LRT line limits our ability to detect true prospective associations between LRT and the outcomes under study. However, self-reported measures are likely to capture commute mode and frequency with sufficient accuracy to provide insight into change over time and the stratified sampling strategy and large sample size allow us to determine whether LRT, sociodemographic, and environmental factors modify change in outcomes. The state-of-the-science objective measures of PA, travel behavior, and built environments, in conjunction with 2-years of follow, strengthen our ability to capture a gradual uptake of LRT use and change in behaviors as the pandemic evolves.

Due to continued COVID-19 surges, the return to on-campus work has been slower than anticipated. As of summer 2022, student instruction and working arrangements remain an in-person and remote hybrid, making the distribution of devices more challenging. Given COVID-19 safety protocols, limited research staff have been working in-office to answer the recruitment phone line, and many measurement appointments have been rescheduled due to participant illness, childcare duties, or changes in staff scheduling. In response, we’ve expanded our device distribution locations so we can meet participants at more convenient campus locations.

Trolley ridership within the San Diego Region is reported at approximately 80% compared to pre-pandemic periods [124]. Multiple factors beyond the introduction of a new LRT line have likely impacted transit usage during the study period, including increased gas prices and changes in transit pass costs. Our surveys and analyses will attempt to capture and explore these factors.

We anticipate difficulty in achieving recruitment goals across income and racial strata as essential workers and people of color have experienced greater stress and negative health impacts due to the pandemic [125] and may have less capacity to participate. We have identified block groups with high concentrations of poverty and people of color to aid recruitment from these areas during the eligibility screening process. We will additionally employ outreach strategies to staff associations and unions representing diverse campus employees. We’ve met with leaders of these organizations to understand members’ concerns (e.g.., parking costs and availability, work schedules) so we can communicate how the study goals align with their interests. We have also formed an Advisory Board of stakeholders, including UCSD administration and staff/student representatives, staff from the City of San Diego, San Diego Association of Governments (SANDAG), and MTS, and representatives from local climate, mobility, and environmental justice advocacy organizations. We will partner with our extensive, interdisciplinary network of stakeholders to ensure the environmental justice and equity goals of the study are achieved, the research is relevant across sectors, and to share data and findings to help inform future investments in San Diego, California, and nationally.

## Supplementary Information

**Additional file 1.** 

## Data Availability

The datasets used and/or analyzed during the current study will be available from the corresponding author on reasonable request.

## Abbreviations

BMI: Body Mass Index
CVD: Cardiovascular disease
QoL: Quality of Life
GPS: Global positioning system
AT: Active transportation
LRT: Light rail transit
MTS: Metropolitan Transit System
MVPA: Moderate-to-vigorous physical activity
PA: Physical activity
SANDAG: San Diego Association of Governments
VMT: Vehicle miles travelled
